# Alternative approaches to managing respiratory tract infections: a survey of public perceptions

**DOI:** 10.3399/BJGPO.2020.0124

**Published:** 2021-03-03

**Authors:** Alex Moore, Rebecca Cannings-John, Christopher C Butler, Cliodna AM McNulty, Nick A Francis

**Affiliations:** 1 School of Medicine, Cardiff University, Cardiff, UK; 2 Centre for Trials Research, Cardiff University Neuadd Meirionnydd, Cardiff, UK; 3 Nuffield Department of Primary Care Health Sciences, University of Oxford, Oxford, UK; 4 Public Health England, Primary Care Unit, Gloucester, UK; 5 School of Primary Care, Population Sciences, and Medical Education, University of Southampton, Southampton, UK

**Keywords:** primary health care, respiratory tract infections, antimicrobial resistance, drug resistance, bacterial

## Abstract

**Background:**

Respiratory tract infections (RTIs) are a common reason for people to consult in primary care, and contribute to antibiotic overuse and antimicrobial resistance (AMR). Alternative approaches to supporting patients with RTIs may help, but it is important to understand public perceptions about these approaches before they are widely implemented.

**Aim:**

To describe public perceptions regarding finger-prick testing, back-up antibiotic prescriptions (BUPs), and alternatives to traditional consultations for RTIs, and identify factors associated with favouring these approaches.

**Design & setting:**

Online national survey (HealthWise Wales) with linked primary care health record data.

**Method:**

Survey item response distributions were described. Associations between responses about consultation alternatives, BUP, and finger-prick point-of-care testing (POCT), and potential explanatory variables, were explored using logistic regression.

**Results:**

A total of 8752 participants completed the survey between 2016 and 2018. The survey found 76.7% (*n* = 3807/4,966) and 71.2% (*n* = 3529/4,953) of responders with valid responses were in favour of being able to consult with a pharmacist or nurse in their GP surgery, or with a community pharmacist, respectively. It also showed 92.8% (*n* = 8034/8659) of responders indicated they would be happy to have a finger-prick test to guide antibiotic prescribing, and 31.8% (*n* = 2746/8646) indicated they would like to be given a BUP if their clinician thought immediate antibiotics were not required. In addition, 47.4% (*n* = 2342/4944) and 42.3% (*n* = 2095/4949) were in favour of having video and email consultations, respectively. Characteristics associated with different response options were identified.

**Conclusion:**

Consulting with pharmacists, using electronic communication tools, and finger-prick testing are widely acceptable approaches. BUP was described as acceptable less often, and is likely to require greater information and support when used.

## How this fits in

Primary care services are struggling to keep up with the demand for RTI consultations, and the pressure to prescribe unnecessary antibiotics is driving antimicrobial resistance (AMR). This study explored public views about alternative approaches to traditional consultations through an online survey. There is scope for incorporating such interventions in current practice, to reduce the strain on GPs, although it is important to ensure adequate information and support are provided.

## Introduction

RTIs are one of the most frequent reasons for consulting in primary care, with 27% of symptomatic people visiting a GP over a 12-month period.^1^ This contributes significantly to GP workload in the UK, which increased by 16% from 2007–2014.^2^

AMR poses a threat to public health, worsens patient outcomes, and increases healthcare costs.^3,4^ Antibiotic consumption drives AMR at both individual and societal levels,^5^ and few new antibiotics are being developed.^6^ Primary care is responsible for 72% of healthcare-related antibiotic prescriptions^7^ and is, therefore, a key priority for tackling AMR.^8^ RTIs accounted for about half of all antibiotic prescriptions in UK primary care between 2013 and 2015.^9^ UK national guidance recommends that GPs do not prescribe antibiotics for most RTIs,^10^ but prescribing continues to be excessive.^11,12^

Alternatives to face-to-face GP consultations may help reduce the pressure on primary care services from RTIs, and use of antibiotics. Non-medical healthcare practitioners, such as pharmacists and nurses, are increasingly managing common infections. Some community pharmacists also provide services such as POCT to help determine the need for GP consultations or antibiotics.^13^ Telephone consultations have been used for years; video and email consultations are becoming more common,^14^ especially during the COVID-19 pandemic.

BUPs, also known as delayed antibiotic prescriptions, reduce antibiotic prescribing and consumption for RTIs, without increasing symptom burden, in clinical trials.^15^ BUPs aim to demedicalise minor infections and cut antibiotic use by empowering patients to share control over their treatment plan.^16,17^ POCT can help reduce antibiotic prescribing for RTIs in primary care.^16,18^ Rapid tests using biomarkers, such as C-reactive protein (CRP), can help reduce uncertainty about the need for antibiotics.^19,20^ GPs report that use of POCT can reduce diagnostic uncertainty, but that costs and the effect on workflow are potential barriers.^21^

This study aimed to explore public perceptions regarding various approaches to consulting for RTIs, and perceptions about BUPs and POCT to target high levels of antibiotic use. Understanding public perceptions may help inform the implementation of different approaches to this challenging problem.

## Method

A questionnaire was adminsitered as part of a national online cohort study in Wales, called HealthWise Wales (HWW).^22^ All adults (aged ≥16 years) who are usually resident or receive their health care in Wales were eligible for inclusion in HWW. Participants were recruited through television, radio, and social media advertising campaigns, as well as promotion through the NHS (hospitals and GP surgeries), pharmacies, large employers, and at cultural events. HWW includes multiple cross-sectional surveys, encompassing general questionnaires and more focused areas of research. The sample consisted of HWW participants who completed a module (questionnaire) called, ‘Caring for Coughs and Colds’ (CCC). The module included 31 questions covering five domains: perceived consulting frequency; drivers of consulting; perceptions of serious illness; alternative sources of information; and views about alternative approaches to consulting for RTIs (Supplementary Table S1). Response options used 5-point Likert scales, ranging from 'strongly agree' to 'strongly disagree' and 'very much in favour' to 'completely against'. Questionnaire development was based on previously published studies;^23^ it used an iterative approach to develop items and a pilot questionnaire to refine items.

### Analysis

Questionnaire responses were summarised using numbers and percentages for categorical variables, and mean and standard deviation for continuous data.

#### Drivers of consulting

After examining response distributions, the 5-point Likert scales for the drivers of consulting and components of a consultation domains were collapsed into binary outcomes of 'agree' (‘strongly agree’ or ‘agree’) and 'do not agree' (‘neither agree nor disagree’, or ‘disagree’, or ‘strongly disagree’) for ease of understanding and to allow for a logistic regression analysis. Multivariable logistic regression analyses were conducted to explore predictors of being in favour of: consulting with a community pharmacist; consulting with a pharmacist or nurse in a general practice; having a video consultation; having an email consultation; receiving a BUP; confidence in knowing when to take a BUP; and having a POCT.

#### Perceived features of serious illness

Response options for the domain on perceived features of serious illness were left as the original, wider 5-point scale ranging from 'very serious' to 'not serious at all'.

#### Alternative sources of advice

The first 3775 participants were mistakenly asked to respond to questions on sources of advice using either 'acceptable' or 'not acceptable'. Unfortunately, it is difficult to interpret these responses, and, therefore, they were not analysed leading to a high level of missing data for these questions. Questions on alternative sources of advice from the remaining responders were recorded using a 5-point Likert scale ranging from 'very much in favour' to 'completely against'. After examining the response distributions, these were collapsed into a three-point scale ('in favour', 'neither in favour nor against', and 'against') and then dichotomised into 'in favour' versus 'not in favour' ('neither in favour nor against' and 'against') and a logistic regression model was conducted to examine characteristics associated with favouring alternative sources of advice.

Data on personal characteristics were obtained from other core HWW modules. Levels of relative deprivation were calculated from home postcode using the Welsh Index of Multiple Deprivation (WIMD), a measure utilised by the Welsh Government.^24^ Consultation and antibiotic prescribing data were obtained through anonymous data linkage with primary care health record data held within the Secure Anonymised Information Linkage (SAIL) Databank. SAIL is an archive for personal, anonymised data, which can be linked on an individual level,^25^ and holds anonymised data for around 80% of Welsh GP surgeries.^26^ Read codes in the primary care record were used to identify consultations for the symptoms of an RTI and antibiotic prescriptions across a 3-year period from January 2015 to December 2017 (Supplementary Tables S2 and S3). SAIL data were also used to identify the presence of Read codes for four key comorbidities (asthma, diabetes mellitus [DM], cardiovascular disease [CVD], and chronic obstructive pulmonary disease [COPD]) (Supplementary Table S4).

For the regression models, the following pre-hypothesised explanatory variables were used: sex; age; deprivation quintile; rurality; current mental health problem; history of mental health problem; marital status; having children; smoking status; reported physical exercise; the four comorbid conditions (asthma, DM, CVD, COPD); consulting and prescribing frequency; and responses to three of the questionnaire items on symptoms reflecting infection seriousness (perception of a cough lasting 1 week or more, perception of a cough with green phlegm lasting 2 days or more, perception of a noisy and wheezy chest). For each analysis, the univariable association between outcome and each independent variable was calculated, and variables associated with outcome at *P* = 0.1 were entered into a multivariable analysis. Results from the models are reported as odds ratios (ORs) with 95% confidence intervals (CIs).

## Results

A total of 8752 participants completed the HWW CCC module between October 2016 and April 2018. Study participant characteristics are presented in Table 1. Just under three-quarters were female, and the mean (standard deviation) age was 49^17^ years. In total, 4387 (51.6%) came from the two most affluent quintiles, and only 12% coming from the most deprived. Less than 10% were current smokers, and just over a third reported that they had not engaged in any exercise in the past week. Responses to questions about alternative sources of health information from the first 3775 (43.1%) participants were discarded because of an error in the response options for these participants. There were no marked differences observed between responders retained in the main analysis and those excluded owing to response options (Supplementary Table S5). A total of 1662 (19%) participants could not be linked to GP data within SAIL.

**Table 1.** Study participant characteristics (*n* = 8752)

Sex	Frequency, *n* (%)
Male	2273 (26.1)
Female	6452 (73.9)
Missing	27
Age, years	
<30	1522 (17.4)
30–64	5512 (63.0)
≥65 years	1718 (19.6)
Missing	0
**Deprivation quintile**	
1 (most deprived)	975 (11.5)
2	1427 (16.8)
3	1710 (20.1)
4	2279 (26.8)
5 (least deprived)	2108 (24.8)
Missing	253
**Rurality**	
Urban settlement >10 000	5130 (60.4)
Town or fringe settlement	1507 (17.7)
Village, hamlet, and isolated dwellings	1862 (21.9)
Missing	253
**Current mental health problem**	
No	6010 (71.8)
Yes	2362 (28.2)
Missing	380
**History of mental illness**	
No	5664 (66.9)
Yes	2800 (33.1)
Missing	288
**Marital status**	
Not married	1940 (30.4)
Married	4445 (69.6)
Missing	2367
**Have children**	
No	2998 (35.8)
Yes	5375 (64.2)
Missing	379
**Smoking status**	
Never smoked	4756 (56.2)
Previous smoker	2890 (34.2)
Current smoker	810 (9.6)
Missing	296
**Physical exercise in the past week**	
None	3107 (36.7)
Some but <1 hour	1197 (14.1)
≥1 hour, but <3 hours	1994 (23.6)
≥3 hours	2166 (25.6)
Missing	288
**Ever diagnosed with asthma**	
No	5926 (83.6)
Yes	1164 (16.4)
Missing^a^	1662
**Ever diagnosed with DM**	
No	6657 (93.9)
Yes	433 (6.1)
Missing^a^	1662
**Ever diagnosed with CVD**	
No	6854 (96.7)
Yes	236 (3.3)
Missing^a^	1662
**Ever diagnosed with COPD**	
No	7032 (99.2)
Yes	58 (0.8)
Missing^a^	1662
**Consulting frequency**	
Never	4866 (71.4)
Less than once a year	1076 (15.8)
About once a year	812 (11.9)
Twice or more per year	65 (1.0)
Missing^a^	1933
**Antibiotic prescribing frequency**	
Less than once a year	5791 (81.7)
Once or more per year	1299 (18.3)
Missing^a^	1662

^a^Owing to non-linkage. COPD = chronic obstructive pulmonary disease. CVD = cardiovascular disease. DM = diabetes mellitus.

**Figure 1.**
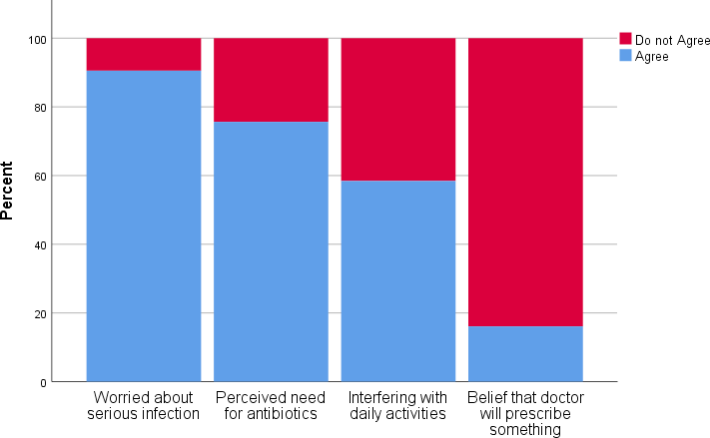
Public perceptions of reasons to consult a healthcare professional for a respiratory tract infection

**Figure 2.**
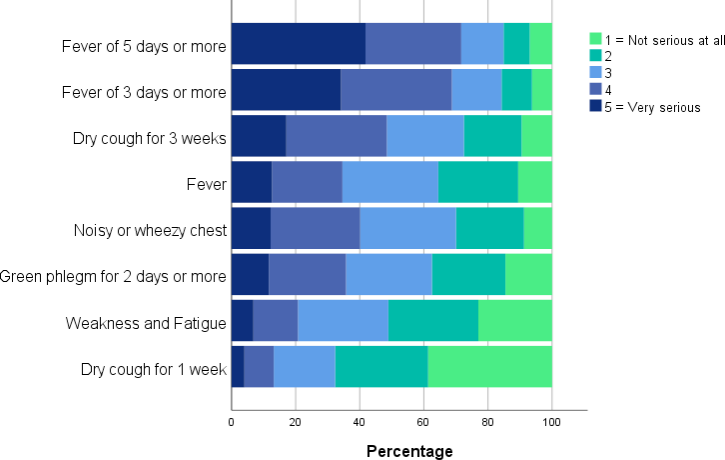
Public perceptions of seriousness of features associated with respiratory tract infections.

The most common reason for deciding to consult about an RTI was worry about an infection being serious (90.5% of participants). However, perceived need for an antibiotic prescription was also an important driver (Figure 1). The illness features most associated with a perception of serious illness were fever, prolonged dry cough (for 3 weeks), noisy and wheezy cough, and green phlegm (Figure 2).

**Figure 3.**
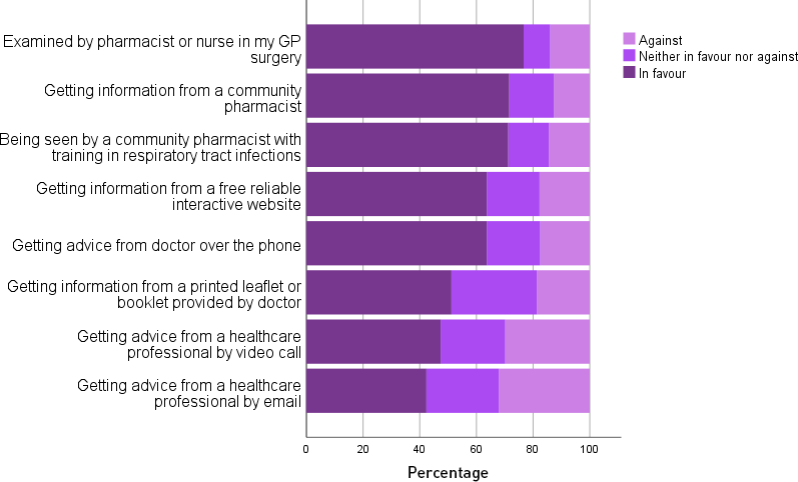
Public views about possible sources of advice or information for respiratory tract infections

Of the 4966 participants who completed a question about consulting for an RTI with a pharmacist or nurse in their usual GP surgery, 76.7% (*n* = 3807) were in favour (Figure 3). Of the 4953 participants who completed the question about consulting with a community pharmacist with training in assessing RTIs, 71.2% (*n* = 3529/4953) were in favour (Figure 3). While 47.4% (*n* = 2342/4944) and 42.3% (*n* = 2095/4949) were in favour of having video and email consultations for RTIs, respectively. It was found that 31.8% (*n* = 2746/8646) and 92.8 (*n* = 8034/8659) were in favour of having a BUP and finger-prick blood tests, respectively. These proportions did not vary significantly by socioeconomic status (Table 2).

**Table 2.** Variation in responses by socioeconomic status

	Deprivation quintile		
Variable	1 most deprived	2	3	4	5 least deprived	Missing	Total
**In favour of:**							
Consulting with pharmacist or nurse in practice	376/498(75.5)	588/783(75.1)	714/920(77.6)	1088/1425(76.4)	918/1187(77.6)	123/153(80.4)	3807/4966(76.7)
Consulting with community pharmacist	347/494(70.2)	556/783(71.0)	650/918(70.8)	1006/1422(70.7)	859/1182(72.7)	111/154(72.1)	3529/4953(71.2)
Video consultation	245/494(49.6)	357/779(45.8)	434/916(47.4)	632/1420(44.5)	607/1184(51.3)	67/151(44.4)	2342/4944(47.4)
Email consultation	220/492(44.7)	325/780(41.7)	377/915(41.2)	613/1421(43.1)	493/1187(41.5)	67/154(43.5)	2095/4949(42.3)
Back-up prescription	351/963(36.4)	469/1411(33.2)	542/1685(32.2)	678/2251(30.1)	624/2085(29.9)	82/251(32.7)	2746/8646(31.8)
Finger-prick blood test	875/958 (91.3)	1276/1416 (90.1)	1572/1690 (93.0)	2110/2257 (93.5)	1966/2087 (94.2)	235/251(93.6)	8034/8659 (92.8)

All results reported as *n/N* (%).

Female sex was associated with being in favour of consulting with a local pharmacist or pharmacist or nurse in their own surgery (Table 3). Adults without children were more likely to favour being seen by a local pharmacist than parents, and individuals who thought that a 1-week cough was serious, a marker of increased concern about RTI symptoms, were more likely to accept a pharmacist or nurse consultation (Table 3). Younger (aged <65 years) and sedentary (no weekly exercise) participants, and those not reporting a mental health problem, were more in favour of video consultations (Table 3). Similarly, younger participants, and those who believed having a wheezy chest is an indicator of serious infection were more likely to favour email consultations.

**Table 3.** Summarised results for the multivariable analysis to identify associations between explanatory and outcome variables

	Localpharmacist^a^	Pharmacist or nurse^a^	Videoconsultation^a^	Email^a^	Receive BUP^b^	Take BUP^b^	POCT^b^
	Adjusted OR^c^ (95% CI)						
**Sex**							
Male (reference)	1.00	1.00			1.00	1.00	1.00
Female	1.48(1.29 to 1.71)	1.46(1.26 to 1.70)			0.80(0.70 to 0.91)	1.18(1.02 to 1.37)	0.64(0.49 to 0.83)
**Age** **, years**							
<30 (reference)	1.00		1.00	1.00	1.00	1.00	1.00
30–64	1.19(0.97 to 1.46)		1.03(0.85 to 1.24)	0.73(0.55 to 0.97)	0.79(0.66 to 0.94)	0.49(0.40 to 0.61)	1.43(1.12 to 1.82)
≥65	1.01(0.80 to 1.29)		0.75(0.59 to 0.96)	0.62(0.44 to 0.88)	0.96(0.77 to 1.20)	0.51(0.39 to 0.68)	1.65(1.16 to 2.35)
**Deprivation quintile**							
1 (most deprived) (reference)			1.00		1.00		1.00
2			0.84(0.65 to 1.08)		0.86 (0.70 to 1.05)		0.80 (0.58 to 1.10)
3			0.88(0.68 to 1.13)		0.83 (0.68 to 1.01)		1.28 (0.91 to 1.80)
4			0.82(0.64 to 1.05)		0.87 (0.72 to 1.05)		1.11 (0.80 to 1.54)
5 (least deprived)			0.99(0.78 to 1.26)		0.86 (0.71 to 1.04)		1.36 (0.97 to 1.90)
**Rurality**							
Urban settlement >10 000 (reference)			1.00	1.00		1.00	
Town or fringe settlement			0.92(0.76 to 1.12)	0.96 (0.76 to 1.21)		1.02 (0.85 to 1.21)	
Village, hamlet, and isolated dwellings			1.03(0.85 to 1.24)	0.90 (0.73 to 1.10)		0.98 (0.83 to 1.16)	
**Current mental health problem**							
No (reference)			1.00		1.00	1.00	1.00
Yes			0.83(0.71 to 0.97)		1.24 (1.09 to 1.41)	1.03 (0.88 to 1.19)	0.94 (0.76 to 1.17)
**History of mental illness**							
No (reference)		1.00					
Yes		1.12(0.97 to 1.30)					
**Marital status**							
Not married (reference)				1.00		1.00	
Married				0.93 (0.76 to 1.15)		1.00 (0.85 to 1.18)	
**Have children**							
No (reference)	1.00			1.00	1.00	1.00	
Yes	0.82(0.70 to 0.95)			1.04 (0.85 to 1.29)	1.17 (1.02 to 1.34)	1.00 (0.85 to 1.18)	
**Smoking status**							
Never smoked (reference)			1.00		1.00	1.00	1.00
Previous smoker			1.09(0.94 to 1.27)		1.08 (0.95 to 1.22)	0.92 (0.80 to 1.06)	0.99 (0.79 to 1.23)
Current smoker			0.83(0.65 to 1.06)		1.17 (0.96 to 1.42)	0.99 (0.78 to 1.25)	0.72 (0.54 to 0.98)
**Physical exercise in the past week**							
None (reference)			1.00	1.00	1.00	1.00	1.00
Some but <1 hour			1.26(1.02 to 1.56)	0.79 (0.61 to 1.02)	0.87 (0.73 to 1.03)	1.18 (0.97 to 1.43)	1.01 (0.76 to 1.36)
≥1 hour but <3 hours			1.20(1.00 to 1.43)	1.13 (0.92 to 1.39)	0.83 (0.72 to 0.97)	0.93 (0.79 to 1.10)	1.01 (0.78 to 1.30)
≥3 hours			1.21(1.01 to 1.44)	1.15 (0.94 to 1.41)	0.81 (0.70 to 0.94)	0.79 (0.66 to 0.93)	1.13 (0.86 to 1.47)
**Ever diagnosed with asthma**							
No (reference)							
Yes							
**Ever diagnosed with DM**							
No (reference)					1.00		
Yes					1.20 (0.95 to 1.50)		
**Ever diagnosed with CVD**							
No (reference)			1.00	1.00	1.00		
Yes			0.84(0.56 to 1.26)	0.67(0.41 to 1.09)	1.19(0.87 to 1.62)		
**Ever diagnosed with COPD**							
No (reference)					1.00		
Yes					2.27(1.21 to 4.27)		
**Consulting frequency**							
Never (reference)				1.00	1.00	1.00	1.00
Less than once a year				1.01(0.81 to 1.26)	1.25(1.07 to 1.46)	0.93(0.78 to 1.11)	1.36(1.02 to 1.82)
About once a year				0.86(0.66 to 1.12)	1.27(1.05 to 1.54)	0.71(0.57 to 0.88)	1.20(0.87 to 1.66)
Twice or more per year				0.88(0.37 to 2.08)	1.32(0.75 to 2.33)	1.36(0.71 to 2.63)	1.34(0.56 to 3.17)
**Antibiotic prescribing frequency**							
Less than once a year (reference)			1.00		1.00		1.00
Once or more per year			0.87(0.69 to 1.10)		1.51(1.23 to 1.85)		0.58(0.42 to 0.80)
**Perception of** **1** **-week** **cough**							
Not serious (reference)		1.00			1.00	1.00	
Serious		1.33(1.08 to 1.65)			1.61(1.37 to 1.89)	0.85(0.70 to 1.02)	
**Perception of green phlegm for** **2** **days**							
Not serious (reference)					1.00		1.00
Serious					1.08(0.95 to 1.23)		0.89(0.71 to 1.11)
**Perception of noisy and wheezy chest**							
Not serious (reference)				1.00	1.00		1.00
Serious				0.85(0.72 to 1.00)	1.24(1.09 to 1.41)		0.86(0.69 1.07)

^a^In favour of consultation; ^b^Agree with use in primary care consultation; ^c^Adjusted for all other factors in the model. DM = diabetes mellitus; CVD = cardiovascular disease; COPD = chronic obstructive pulmonary disease.

### Back-up prescriptions and finger-prick blood tests

A total of 2746 (31.8%) agreed they would like to be given a BUP if their clinician thought they did not need immediate antibiotics. While 2922 (33.8%) responders indicated that they would not feel comfortable in knowing when to take a BUP. There was an association between socioeconomic deprivation and wanting to be given a BUP, with those from more deprived backgrounds being more in favour of this approach (Table 2). However, in a multivariable analysis socioeconomic status was not significant. Those who reported that they would be happy to receive a BUP being more likely to be male, young, have a current mental health problem, be a parent and be sedentary (report not engaging in any exercise). A history of COPD was associated with twice the odds of wanting to be given a BUP. Consulting less frequently, receiving antibiotics more frequently, and having greater concern about symptoms (believing that a 1-week cough and a noisy, wheezy chest are indicators of serious infection) were all associated with a greater odds of wanting to be offered a BUP. Women, younger adults (aged <30 years), those who are more sedentary and those who consulted less frequently, were all more likely to indicate that they would not feel comfortable in knowing when to take a BUP if they were given one (Table 3).

A total of 8034 (92.8%) participants agreed that they would generally be happy to be offered a finger-prick blood test to help guide antibiotic treatment during a consultation. Women, younger people, smokers (current and previous) and frequent antibiotic recipients were less likely to report finger-prick blood testing acceptable (Table 3).

## Discussion

### Summary

Key findings in this survey of public perceptions about consulting for RTIs are that more than 90% of responders indicated that they would be happy to have a finger-prick blood test in primary care to help inform the need for antibiotics, and around three-quarters would be happy to consult with a community pharmacist, nurse, or pharmacist in their surgery. Women, adults without children, and people with greater concern about the seriousness of cough, were more in favour of having the option of consulting with non-medical practitioners. Less than half of responders thought electronic consultations (video or email) were acceptable, with younger people being more in favour of this approach. Less than a third of participants indicated they would want to be given a BUP, and a third said they would not feel comfortable deciding when to use a BUP.

### Strengths and limitations

The study benefited from being able to link perceptions reported in an online survey to primary care health record data. Data were obtained from over 8700 people; however, the study had over-representation of females (73.9% female compared with 50.7% of the UK population),^27^ and middle-aged (30–64-year-old age group) participants. The survey was only available to those with internet access between October 2016 and April 2018, which was 85% of Welsh households in 2018.^28^ The responders were on average less socioeconomically deprived than the general population, which is likely why the study had a lower proportion of current smokers than in the general population. Nevertheless, the authors were able to include large numbers from all socioeconomic groups and control for socioeconomic status in the analyses. An association was found between socioeconomic status and views about use of BUP, but this was not significant in a multivariable analysis, and no other significant associations were found with socioeconomic status. A total of 3775 (43%) of the sample was lost owing to a mistake with response outcomes; however, the excluded samples and those retained were broadly similar and appears to exhibit little bias. Finally, views expressed in an online survey may differ from those expressed in other settings or following greater provision of information. For example, questions about BUP may have differed if responders had a more detailed understanding of the reasons for using this approach and how it works.

### Comparison with existing literature

How people appraise their symptoms and perceive the severity of their illness was identified as a key driver of consulting in a previous qualitative study and survey of adults across England.^23^ Women were more likely to consult, but there were no differences by age or region in this study. Worry about cough has previously been shown as a driver of consulting in general practice.^29^ It was found that those who perceived a 1-week cough as serious were more likely to indicate that consulting with a pharmacist would be acceptable. This suggests that those with greater concern about RTI symptoms want different consultation options, but this needs confirming.

A 2014 public survey found a similar proportion (just over a third favouring or strongly favouring) BUP or delayed prescriptions, as in the present study (31.8%).^30^ Their findings are consistent with the present study's finding that women and older people are less likely to favour delayed or BUP antibiotics, but they also found that women and parents were more aware of BUPs.^31^ Perceptions about deciding when to use BUP antibiotics were also explored and it was found that a third were not comfortable making this decision. A previous qualitative interview study found that most patients were happy to be given BUP, but some found it confusing to be issued a prescription after being told that it was viral.^32^ A similar study in Australia identified concerns about the use of BUP, and, in particular, knowing when to take them.^33^ GPs have expressed mixed views about BUPs.^34,35^

### Implications for research and practice

The findings support the view that several approaches to managing RTIs are likely to be acceptable to the public. Pharmacists, nurses, and allied health professionals already play an important role in managing RTIs, and the findings suggest that many value these options, with men and parents reporting less acceptance. These findings may help those designing services, and further research could explore the reasons for these differences. Less enthusiasm was found for digital (video and email) consultations for RTIs. Previous qualitative research has found that this approach is generally very acceptable to patients, although technical issues can be a barrier to use.^36,37^ In the context of the COVID-19 pandemic, remote consultations have become the norm, and it would be interesting to see how public perceptions on use of these technologies have changed.

It is encouraging to see that more than 90% were happy to have a finger-prick blood test to help guide antibiotic prescribing. POCT can improve antibiotic prescribing for RTIs,^16^ including acute exacerbations of COPD.^38^ The findings suggest that members of the public have mixed views about the role of BUPs. There is clearly a need for more public information about BUPs, including a rationale for use of BUP and clear instructions on when to initiate them. These findings can be used to help develop strategies to improve the management of RTI and reduce the use of antibiotics in primary care.
